# Notes on Tuberculosis—Avenues of Invasion

**Published:** 1896-12

**Authors:** Jas. T. Whittaker

**Affiliations:** Cincinnati


### SELECTIONS.

### Notes on Tuberculosis—Avenues of Invasion.

BY JAS. T. WHITTAKER, M.D., CINCINNATI.

*Tuberculosis of the Alveolar Processes.* Tuberculosis of the alveolar processes has been known for twenty-five years, and thirty-seven cases have been reported up to the present time. Zaudy makes a perfect description of the condition, emphasizing the fact that the teeth, especially when affected with caries, constitute an orifice of entrance for the tubercle bacillus. The wounds left after the extraction of teeth are also subject to infection by sputum containing the bacillus.

It would appear that tuberculosis of the alveolar processes may be primary—it never remains long isolated, but extends to the vicinity. Points of predilection may not be determined. The disease occurs most frequently between fifteen and fifty years, and in men oftener than in women. Any distinction be-

* Cosmos, Sept., 1895.

† Dental Review, March, 1895.

tween lupus and tuberculous infection is not practicable; the lighter, more superficial forms are usually ascribed to lupus, while the deeper, graver forms, which lead to necrosis of bone, are ascribed to tuberculosis.

The condition is scarcely to be confounded with carcinoma, perhaps more frequently with syphilis. The literature shows that in tuberculosis of the alveolar processes there are numerous cases in which the bacillus remains undemonstrable, notwithstanding every study. In the case reported by the author, the bacillus could not be disclosed in the granulation tissue, notwithstanding the fact that miliary tubercles and giant cells, with nuclei at the borders, could be readily demonstrated.

